# 3D brain tumor segmentation using an improved V-Net architecture and 3D attention gate

**DOI:** 10.1016/j.ynirp.2026.100364

**Published:** 2026-06-08

**Authors:** Sima Esmaeilzadeh Asl, Mehdi Chehel Amirani, Hadi Seyedarabi

**Affiliations:** a Department of Electrical Engineering, Urmia University, Urmia, Iran; b Faculty of Electrical and Computer Engineering, University of Tabriz, Tabriz, Iran

**Keywords:** V-net, Attention gate, 3D brain MRI, Deep learning, Tumor segmentation

## Abstract

Tumor segmentation involves the identification and definition of tumors in medical images, which is crucial for cancer diagnosis and treatment as it enables accurate measurement of the size and shape of the tumor. This can be done manually or automatically with computer algorithms. Automated segmentation reduces the time and effort of manual methods while enhancing accuracy and stability. This article uses BraTS2021 data for 3D brain tumor segmentation. Initially, an N4 BiasField Correction Filter preprocesses MRI data. An improved V-Net is used for segmenting enhanced tumors, whole tumors, and tumor cores. The V-Net processes image data in three dimensions, with enhancements in the decoder and encoder sections, improving overall performance. The encoder benefits from residual and dilated convolution layer, while the Attention Gate enhances the decoder. In the encoder, the dilated convolution layer enhances performance, while residual layers ensure that adding more layers doesn't harm the network. All four MRI types are input into the 3D improved V-Net, achieving Dice coefficients of 87%, 81.2%, and 74.43% for whole tumors, tumor cores, and enhanced tumors respectively. Results indicate this method effectively segments 3D brain MRI images.

## Highlights

• Using attention mechanisms that focus on relevant features while suppressing irrelevant information.
• Employing dilated convolution to enable the network to capture more contextual information from input data without losing resolution.
• Using residual blocks to facilitate the training of deeper networks effectively, prevent vanishing gradients, promote feature reuse.
• Implementing V-Net to generate volumetric segmentation maps, where each voxel is classified into a specific category.
• Improving tumor segmentation accuracy, helping treatment planning and monitoring for better patient outcomes.

## 1 Introduction

Nowadays, brain tumors have been established as an illness that can even lead to death. Brain tumors can interfere as they are the result of abnormal cell growth within and around the central nervous organs which might also apply extra pressure on the protective tissue surrounding the brain (Khaled Abd-EllahAli et al., 2016). Additionally, it has also been noted that most of these tumors occur even in people who do not have any identified risk factors. It is noted that although anyone of any age can develop a brain tumor, Individuals aged undre 20 or over 60 years are more likely to have brain tumors. Brain tumors can be classified as benign (non-cancerous) or malignant (cancerous) (Li et al., 2020). However, in either case, the tumor itself presents a distinct challenge to the patient. Benign tumors are not dangerous because they do not metastasize to sudden tissues or other regions of the body. Malignant tumors are cancers that can spread to other parts of the human anatomy. Early diagnosis of brain tumors can reduce the amount rate of fatalities from brain tumors (Dora et al., 2018). Brain tumors like all other tumors can cause a variety of signs, however, their pattern may vary depending on the exact type of brain tumor, its localization within the brain, and the speed of the tumor growth. In today's age of innovations, the application of electromagnetic signals and advanced medical imaging techniques, such as CT scans and MRIs, has generated a vast amount of data regarding human brain anatomy. This information is crucial for evaluating and planning patient treatments; however, manually interpreting such large data is nearly impossible. Consequently, there is a crucial need for automated and efficient methods to aim in treatment decision-making. Recent scientific advancements in medical image processing have significantly improved the precision of tumor area diagnoses and have a wide range of applications in disease detection and treatment design. Ultimately, the image produced through these methodologies evaluated healthcare professionals in accurately diagnosing brain tumors (Gilanie et al., 2017).

MRI provides crucial information about the shape, size, and location of brain tissue while avoiding the high levels of ionizing radiation associated with other imaging techniques. This method is capable of getting images of both hard and soft tissues, typically beginning with one or more scans. Modern devices are designed to generate images in three plans: Coronal, sagittal, and axial. Various pulse sequences are employed to enhance contrast and facilitate tumor detection. The automatic segmentation of brain tumors faces numerous challenges, and as deep learning technology progresses, these difficulties have become increasingly complicated. Currently, various algorithms, methods, and techniques are used to predict and classify tumor regions in brain images.

2D approaches inherently fail to preserve the spatial continuity across image slices, thereby losing critical inter-slice contextual information. Conversely, conventional 3D methods often struggle with the computational burden imposed by high-volume data, which can limit their effectiveness in accurately delineating the boundaries of heterogeneous tumors. To address these challenges, we integrate attention gates with dilated convolutions, enabling the network to expand its receptive field without increasing the number of parameters, while simultaneously emphasizing important regions within the data.

Xiangyu Li et al. (2020) used a multi-stage cascaded (hierarchical) network to detect tumors and their components. In the segmentation process, the outputs from previous stages are used as inputs for the subsequent stages. To avoid overfitting, several auxiliary outputs are included for monitoring in the next steps. This hierarchical architecture uses ResNet, including blocks like squeeze-and-excitation after each convolution block and concatenation, specifically designed for tumor segmentation. Lingraj Dora et al. (2018), presented a new hybrid approach that used a Gauss-Newton-based algorithm (GNRBA) alongside a feature selection technique. Research in this area has achieved accuracy, sensitivity, specificity, and ROC curve as quantitative metrics for evaluating the performance of these algorithms. Chenggang Lyu et al. (Lyu and Shu, 2020) introduced a two-stage encoder-decoder model for automatic brain tumor segmentation that uses a series of two networks. The first encoder uses ResNet blocks to process the input data. The input of the second encoder is generated from the segmentation map extracted by the first stage, providing for improved accuracy in the segmentation process. Fabian Isensee et al. (2021), evaluate brain tumor segmentation by including preprocessing modifications to the BraTS2020 datasets, using data augmentation techniques, and applying region-based training methods. Additionally, several minor adjustments to the 3D U-Net, known as nnU-Net, are used to enhance image feature extraction, ultimately leading to improved segmentation performance.

Due to the lack of clarity in the third dimension of brain images in the BraTS dataset, image segmentation is performed from the axial view, as observed in Havaei et al. (2017). In the model of this paper, each two-dimensional axial slice is processed using different MRI sequences. Saddam Hussain et al. (2017) investigated the efficacy of convolutional neural networks in brain segmentation, consisting of three steps: pre-processing, CNN application, and post-processing. After pre-processing, input images are divided into segments and fed into a convolutional neural network to predict labels for each segment. Feifan Wang et al. (2020) used 3D U-Net segment brain images by using normalization and image patching techniques that are optimized for the low-power GPUs used in this paper. Minglin Chen et al. (2020) used a multi-scale prediction method from the decoder of the 3D network, using different resolutions. The final prediction selects the minimum pixel value from multi-scale upsampling. M Hamghalam et al. (2020) have implemented a trained convolutional neural network, such as a GAN, to produce high-contrast images that modify the intensity distribution of brain lesions within internal subregions. This approach uses the 3D FCN architecture for segmenting images from the BraTS2019 dataset.

Minh H et al. (Vu et al., 2020) proposed a hierarchical architecture that uses ResNet and combined squeeze-and-excitation blocks after each convolution layer, along with concatenation, specifically designed for tumor segmentation. Soopil Kim et al. (2020) employed a specific U-Net architecture to detect tumors using two-dimensional brain images taken at three different levels. In the initial step, four-channel two-dimensional brain images serve as input to the network. Finally, a Random Forest algorithm is employed for survival prediction. M Amian et al. (2020) described an architecture that consists of two parallel paths: the first focuses on learning local features of the input data, while the second provides a total view of the whole images. The outputs from both paths are combined to create a comprehensive training set of images. Rehan R et al. (Rehan et al., 2023) used a combination of U-Net and ResNet for brain tumor segmentation. In the encoder section of the network, residual connections are used, while the decoder uses the capabilities of U-Net. The 3D BTS network proposed by Zhu et al. in (Zhu et al., 2024) named SDV-TUNet (Sparse Dynamic Volume TransUNet), features an encoder-decoder structure that efficiently uses volumetric data to enhance segmentation accuracy. This network processes volumetric data using a 3D framework with enhanced depth modeling for dense predictions. It has two main modules: the sparse dynamic (SD) encoder-decoder module and the multi-level edge feature fusion (MEFF) module. The adaptive fusion strategy with the shifting window in 3D has enabled dynamic recognition of regional connectivity and interaction between multi-axis information through both local tight and long-range sparse correlations during encoding for global spatial semantic features. Yousef R et al. (Yousef et al., 2023) introduced a U-Net structure known as “Bridged U-Net-ASPP-EVO,” featuring two models specifically designed for enhancing brain tumor segmentation. This model uses “Atrous Spatial Pyramid Pooling”, which is required for tumor segmentation of different sizes. It achieved impressive average segmentation Dice scores on the BraTS2021 validation dataset. Most of the existing methods directly extract global semantic features from deep spatial dimensions in 3D volumes, which often fail to get voxel information, interlayer connections, and adequate details. Liang et al. (2023) introduced a 3D U-shaped architecture called Swin-Unet, a symmetrical segmentation network for brain tumor analysis, drawing inspiration from the Swin Transformer architecture. This model integrates the strengths of the 3D Swin Transformer into both the encoder and decoder of the segmentation network. To improve performance, a self-supervised learning framework was used for training the encoder, which requires a detailed reconstruction process that adapts the behavior of the model. However, the combination of CNN with the Swin Transformer feature layer at a high-resolution filtering level may limit the ability of the model to get local semantic features, which leads to a similar phenomenon as fine-tuning and lower segmentation performance in some areas. Esmaeilzadeh et al. (Esmaeilzadeh Asl et al., 2023) utilized a two-dimensional U-Net for analyzing 3D MRI analysis in the BraTS2020 dataset. The MRIs are captured in 2D slices at three different levels. For pre-processing, a combination of bilateral and Black-hat filters is applied. Subsequently, a 2D U-Net is utilized for processing the 2D MRI images, with a median filter implemented to improve images.

Deep learning is a subfield of machine learning and has evolved to be used for feature extraction and segmentation in brain MRI. This has greatly improved the correct diagnosis of brain tumors. However, a research gap remains, especially with 3D models. However, U-Net and its variants are widely used methods in brain tumor segmentation. In this article, we develop a new architecture of the V-Net that stands out by using dilated convolution, residual layers, or Attention Gates. This approach provides an effective and automatic method for accurate segment bordered by the tumor itself. Current 2D segmentation methods for brain MRI data often fail to capture the full spatial context available in three-dimensional (3D) volumetric images. To address this, our approach uses a novel 3D V-Net architecture enhanced with a specialized 3D Attention Gate to better use the 3D structure of the data. This attention mechanism selectively emphasizes relevant features across all dimensions, depth, height, and width, enabling the network to focus on critical tumor regions while reducing the influence of irrelevant background areas. To support training deeper networks, we include residual layers that enable smoother gradient flow through identity connections, which helps preserve important low-level features and improves training stability. Additionally, we employ dilated convolutions to expand the network's receptive field, allowing it to capture diverse tumor features at multiple scales without increasing computational demands or sacrificing image resolution. Together, these elements work to provide a more accurate and robust segmentation of complex brain tumors in volumetric MRI scans.

We propose an enhanced three-dimensional V-Net architecture that incorporates residual connections alongside dilated convolutions to improve feature preservation and ensure stable training. In addition, a three-dimensional attention gating mechanism is employed to dynamically weight features across all spatial dimensions (height, width, and depth) thereby enhancing the model's representational capacity. Compared to existing approaches, the proposed framework also reduces dependence on extensive preprocessing steps, contributing to a more efficient and streamlined workflow.

Dilated convolutions are employed to capture broader contextual information from tumor regions, whereas residual connections enhance gradient flow and contribute to more stable training. Additionally, the attention gating mechanism leverages decoder signals to suppress irrelevant features from the encoder and to emphasize regions of interest, such as tumor core and edema, thereby improving the network's focus on clinically significant structures.

**Fig. 1:**
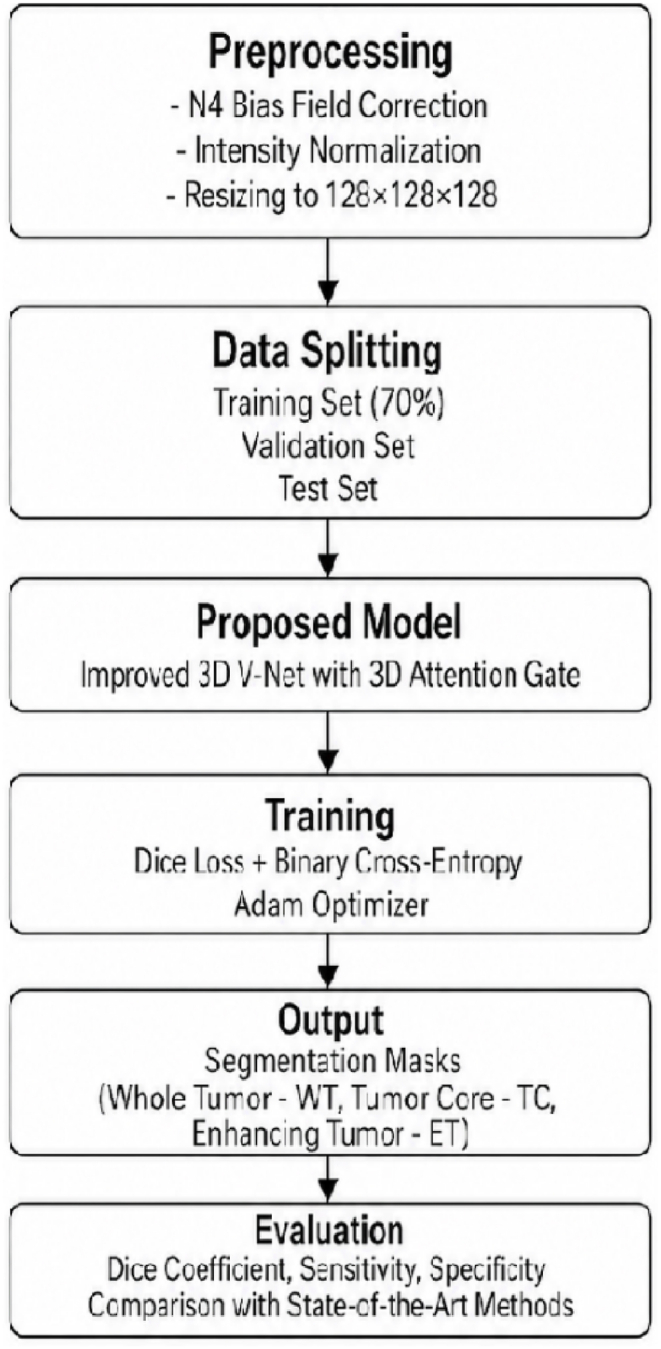
The overall pipeline of the proposed 3D brain tumor segmentation method.

First, the original image was preprocessed. Next, a new segmentation method for tumor detection is explained with a detailed V-Net architecture. The data from tumor images helps in understanding the details to be segmented. The last part directs the results yielded by the model. Fig. 1 provides a detailed pipeline of our work.

## 2 Materials and methods

### 2.1 Dataset

The proposed method is evaluated on the BraTS2021 dataset, which includes MRI sequences from 1251 patients. Two categories of brain tumors are in this dataset: High-Grade Glioblastoma (HGG) and Low-Grade Glioblastoma (LGG). Each patient has four images across four channels: T1, T1c, FLAIR, and T2, with each image sized at 240 × 240 × 155. All scans are available as NIfTI files (.nii.gz). For each patient, a mask is provided that contains four classes, i.e., 1 for necrosis tumor core, 2 for edema, 4 for enhancing tumor, and 0 for background. Ground truth is manually annotated by experts. The task of the model is to detect and segment three tumor regions: the whole tumor, tumor core, and tumor enhancement. The dataset is split into 70% (876 patients) for training and the remaining 30% of the data will be used as a validation and test set. The original images have been resized to 128 × 128 × 128.

### 2.2 Data pre-processing

**Fig. 2:**
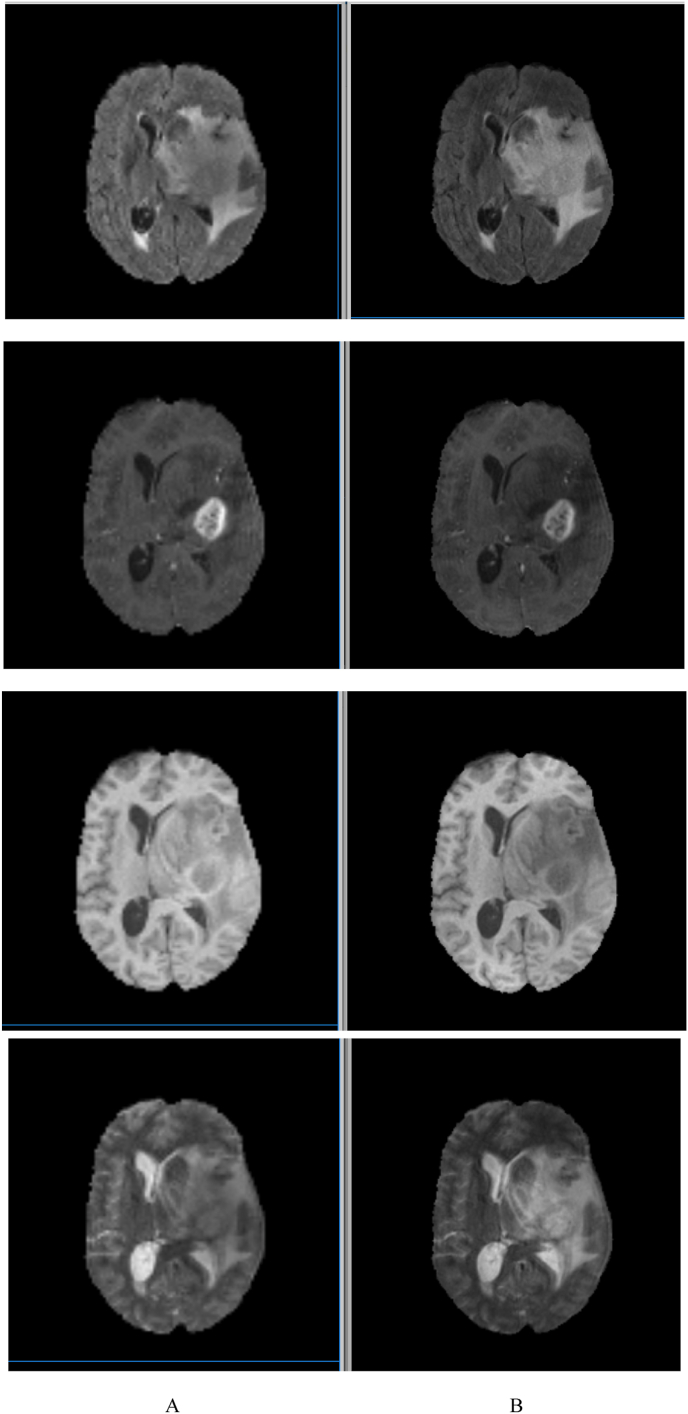
The pre-processed image is displayed alongside the raw image from Brain MRI across four different scan types. A) The processed image, B) The raw MRI image.

Changes in the magnetic field of MRI scanners cause bias field disturbances that can affect image quality. These bias fields cause low-frequency noise, which can uncover high-frequency details, which resulting in less defined edges and lines in the MRI images (Juntu et al., 2005). To address this issue, the non-parametric non-uniform intensity normalization algorithm (N3) for bias field correction is employed for its efficiency and versatility in various applications, combined with public access, have made it a widely used tool for a long time (Vovk et al., 2007). The N3 algorithm works iteratively by modeling the bias field through B-spline the least squares fitting. In (Vovk et al., 2007), an enhanced N4 bias field correction method is introduced, using the filter ITK: N4BiasFieldCorrectionImageFilter It is an enhancement to the N3 correction available in ITK 5.0.0. This improvement is especially important for the improved B-spline approximation strategy, which accelerates execution time. This strategy involves parallelizing the B-spline estimation algorithm and using a multi-resolution method to estimate the remaining bias area during each iteration. Fig. 2 shows the original and the pre-processed version of the MRI image.

### 2.3 Proposed methodology

As computer vision technology advances, variable convolutional networks are increasingly being used in medical image analysis. Although convolutional networks are good at processing 2D images, most clinical medical data is in 3D format.

Although many experts and researchers have recommended various deep learning network architectures, this provides potential results in brain tumor segmentation. However, the essential dissimilarity of high-grade MRI images of brain glial tumors presents significant challenges. This variability is evident in the variation and irregular shapes of the tumors. Deep learning segmentation methods typically require large datasets; however, information about brain tumors is often limited and complex. High diversity has significant differences between sub-regions of the tumor. This complicates the distinction between tumor and non-tumor areas. These factors interfere with the accuracy of brain tumor segmentation.

In this study, we address critical limitations in current brain tumor segmentation models, particularly the challenges presented by tumor heterogeneity and the underutilization of three-dimensional (3D) spatial information. To overcome these issues, we propose a novel hybrid architecture that integrates a 3D Attention Gate module into the V-Net framework. Unlike conventional approaches that often prioritize limited regions of an image, this module dynamically weights volumetric features across all three spatial dimensions, length, width, and height, enabling enhanced focus on tumor boundaries. This capability is particularly vital for getting the irregular and complex geometries of tumors, as exemplified in the BraTS2021dataset.

To support robust and stable training of deeper networks, our architecture incorporates residual layers, which facilitate effective gradient flow and preserve essential low-level information, thereby preventing degradation or loss of critical features. Additionally, we employ dilated convolutions to expand the network's receptive field without increasing computational complexity or compromising image resolution. This allows the model to simultaneously capture fine details and broader structural patterns, enhancing its ability to analyze diverse tumor characteristics.

The synergistic integration of these components, the 3D Attention Gate for precise feature prioritization, residual layers for sustained information flow, and dilated convolutions for an expanded contextual view, significantly improves the model's performance. Collectively, these advancements enable more accurate and comprehensive segmentation of complex brain tumors, offering a promising approach for advancing diagnostic precision in medical imaging.

**Fig. 3:**
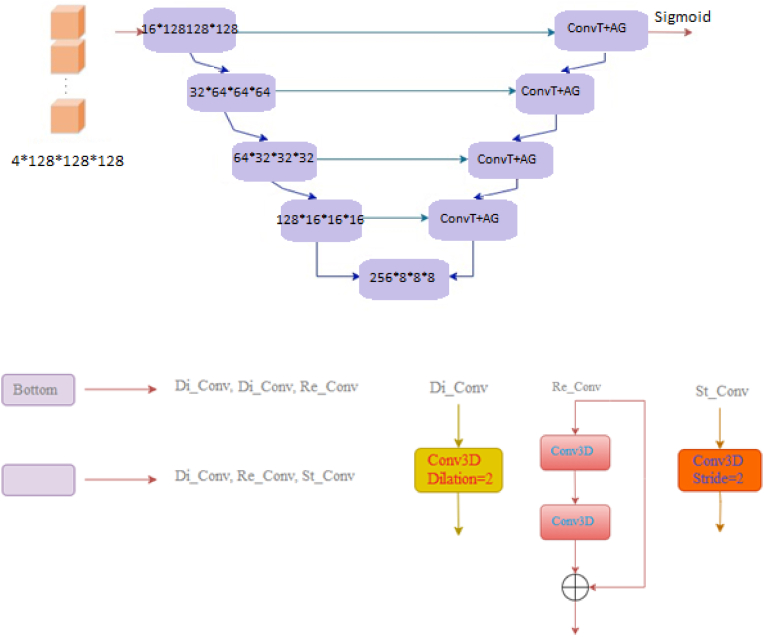
The overview of the structure of the proposed V-Net model for 3D brain tumor segmentation, illustrating the encoding path with dilated convolutions and residual blocks, and the decoding path with deconvolution and skip connections and 3D Attention gate.

Fig. 3 shows the architecture of our proposed V-Net model, used for 3D segmentation of brain tumors using multimodal MRI data from the BraTS2021 dataset. The model adopts a symmetric U-shaped structure, including an encoding path and a decoding path, to enable end-to-end volumetric segmentation. This design facilitates the direct processing of 3D MRI volumes, producing accurate segmentation outputs without requiring intermediate fully connected layers. The V-Net inputs the data into the model as volume input and uses 3D convolution for processing the MRI images.

Concatenation-based skip connections are utilized to directly fuse low-level features from the encoder with high-level representations in the decoder. This strategy facilitates the preservation of fine spatial details and improves feature reuse across the network. On the right side, the decoder decompresses the signal until it returns to its original dimensions.

Encoding path: The encoding path extracts hierarchical features from input MRI volumes through a series of 3D convolutional operations, organized into multiple steps that progressively reduce spatial resolution while getting both local and global contextual information. Each step consists of three key components designed to optimize feature extraction:

1. Dilated Convolution Layer: This first layer uses 3D dilated convolution with a dilation rate of 2, enabling the network to expand receptive field to get spatially distributed tumor features across a larger input volume. By increasing the contextual features without increasing the parameter size, this approach enhances the model's ability to detect complex tumor patterns in 3D MRI data.
2. Residual Block: Each step includes a residual block composed of two sequential 3D convolutional layers with skip connections. These connections allow the input to feed forward layers and directly feed into subsequent steps, reducing the vanishing gradient problem and ensuring stable training of deeper networks. Nonlinear activation via Parametric ReLU (PReLU) is applied, and the residual output is computed by adding the input to the final convolutional layer's output, preserving critical low-level features.
3. Downsampling Layer: A 3D convolutional layer with a stride of 2 reduces the feature map resolution using 2 × 2 × 2 kernels, halving the feature map size at each step. This operation replaces traditional pooling layers, offering controlled feature extraction while avoiding information loss. Additionally, the number of feature channels is doubled, deepening the feature representation.

All convolutional layers in the encoding path use 5 × 5 × 5 kernels to maintain a consistent receptive field, with PReLU activation introducing nonlinearity to capture intricate patterns.

To accommodate memory constraints during training, convolutional layers may substitute pooling operations, eliminating the need for backpropagation switches. This design ensures efficient downsampling while enriching feature complexity, with each step generating twice as many feature channels as the previous one, forming a robust feature map for the decoding path.

Decoding path: The decoding path operates symmetrically to the encoding path, aiming to restore the original spatial resolution of the input volume while preserving the extracted features to produce a segmentation map. It includes steps that progressively upsample low-resolution feature maps, culminating in a two-channel output distinguishing tumor and background regions.

Each decoding step begins with a 3D convolutionTranspose layer that upsamples the feature maps using kernels to increase spatial dimensions. This is followed by one to three convolutional layers, each employing half the number of 5 × 5 × 5 kernels compared to the corresponding encoding stage. These layers refine features and ensure accurate recovery of spatial details.

Horizontal skip connections, as shown in Fig. 3, transfer high-resolution features from the encoding path to corresponding decoding steps. These connections compensate for spatial information lost during downsampling, significantly enhancing segmentation accuracy and accelerating model convergence by providing contextual information to early decoding layers.

The architecture is finished with a 1 × 1 × 1 convolutional layer that computes a feature map to the input volume's dimensions, producing a two-channel output. A sigmoid activation function is applied to generate a probabilistic segmentation map, classifying each voxel as belonging to tumor subregions (e.g., enhancing tumor [ET], whole tumor [WT], tumor core [TC]) or background.

The proposed V-Net architecture uses its end-to-end training capability to process 3D MRI volumes directly as input, and produce detailed segmentation outputs. The integration of dilated convolutions, residual blocks, and skip connections balances the get of global context with the preservation of local details, while deconvolution operations restore volumetric integrity. As validated with the BraTS2021 dataset, this design effectively adapts to the complex and irregular geometries of brain tumors, achieving robust segmentation performance. Fig. 3 provides a schematic overview of these components, highlighting the model's structural coherence and functional efficacy.

#### 2.3.1 Attention gate

**Fig. 4:**
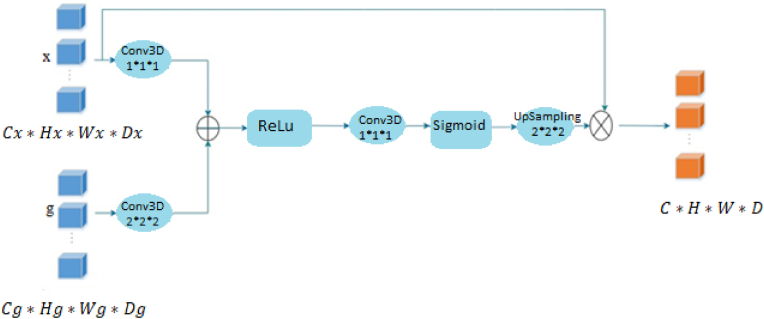
Structure of Attention Gate model that enhances feature relevance across volumetric data.

To enhance the accuracy of brain tumor segmentation in 3D MRI volumes, such as those in the BraTS2021 dataset, we propose combining a 3D Attention Gate module into the V-Net architecture, drawing inspiration from the Attention mechanisms in (Oktay et al., 2018). This module enables the model to focus on important tumor regions while suppressing irrelevant background noise, thereby improving segmentation accuracy. The 3D attention gate is strategically applied before each skip connection in the V-Net, dynamically weighting features to those most relevant to the task. This section explains the implementation, operation, and impact of the 3D Attention Gate on segmentation performance. As shown in Fig. 4, the 3D attention gate receives two input feature maps: the feature map ***x*** from the encoding path via skip connection, containing high-resolution features from earlier network layers. Gating signal ***g*** is obtained from the processing layer on the decoding path, providing deeper, context-rich features. To align these inputs, their channel dimensions are coordinated through convolutional operations. A 1 × 1 × 1 convolution is applied to ***x,*** reducing its channel count while preserving spatial details. A 2 × 2 × 2 convolution with a stride 2 is applied to ***g***, adjusting its spatial resolution to match that of ***x*** with 16 filter channel. Both inputs are normalized with GroupNormalization (8) to remain train stability. The result of feature maps are summed along the channel dimension, and combine local details ***x*** with global context from ***g.*** This combination feature map is processed through the ReLU activation function to enhance the detection of complex patterns. Next, the output is passed through an upsampling layer using another 1 × 1 × 1 convolution layer followed by a sigmoid activation function. This process provides attention weight coefficients, denoted as αᵢ ∈ [0, 1], which indicate the importance of each voxel in the image. The learning rate for whole V-Net architecture, which includes the 3D Attention Gate module, is adjusted using Adam optimization. We begin with an initial learning rate of 0.01 and momentum coefficient of 0.99. This learning rate is halved every 10 epochs, as detailed in Section 3. The final output of the 3D attention gate is computed by multiplying the input feature map ***x*** with these attention coefficients:

(1) $${\hat{x}}_{i,c}^{l}=x_{i,c}^{l}.\alpha_{i}^{l}$$

In the default setting, a single scalar attention coefficient is computed for each spatial location (voxel) and applied across all channels. $N_{l}$ denotes the number of feature maps (channels) in layer l, i.e., c ∈ {1, …, N }. Adding weight information to the input feature map of this layer can help remove unrelated data in the skip connection. As shown in Fig. 4, the output of the 3D attention gate is multiplied with the encoder feature to include context information:

(2) $$\left\{ \begin{array}{c} C=Cx+Cg\\ H=Hx=Hg\\ W=Wx=Wg\\ D=Dx=Dg \end{array}\right.$$

Where C, H, W, and D represent the channel, height, width, and depth dimensions of the feature maps. The subscripts xxx and ggg denote the input feature map and gating signal, respectively. Eq. (2) ensures that both inputs share identical spatial and channel dimensions for subsequent attention computation.

The 3D Attention Gate module improves the V-Net's segmentation performance by focusing on tumor regions (e.g., enhancing tumor [ET], whole tumor [WT], tumor core [TC] in BraTS2021) while minimizing background interference. Unlike traditional approaches that may unintentionally increase irrelevant features, this module offers several advantages:

Dynamic feature weighting enables accurate depiction of complex tumor boundaries, critical for irregular geometries. By suppressing irrelevant regions, the module enhances the quality of segmentation maps and reduces noise. Skip connection with filtered features to evaluate and stabilize network training.

Validation of the BraTS2021 dataset demonstrates that the 3D Attention Gate improves the Dice Score by 3-5% for complex tumors, reflecting the model's ability to get multiscale and irregular tumor structures. Compared to 2D Attention Gate mechanisms, the 3D approach fully uses volumetric context, ensuring comprehensive feature prioritization across all spatial dimensions.

### 2.4 Performance metrics

To evaluate the segmentation performance of the 3D model, we used the BraTS2021 dataset and the following evaluation criteria: Dice coefficient, sensitivity, and specificity.

The Dice coefficient is defined as follows:

(3) $$Dice\;core=\frac{2.\left|A\cap B\right|}{2.\left|A\cap B\right|+\left|B\backslash A\right|+\left|A\backslash B\right|}$$

Sensitivity and specificity are also defined, where TP is true positive, FP is false positive, TN is true negative, and FN is false negative, indicating the number of each type of voxel:

(4) $$Sensitivit=\frac{TP}{TP+FN}$$

(5) $$Specificity=\frac{TN}{TN+FP}$$

## 3 Results

**Fig. 5:**
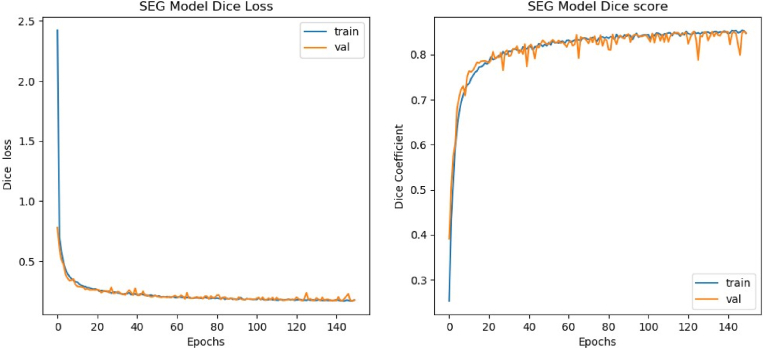
Dice Coefficient and Loss plot for training and validation data.

In this article, an attention gate module is integrated into the encoder of the V-Net network, which consists of different blocks. This module takes advantage of channel interdependence to learn spatial weight information and feature maps by detecting structure of correlated regions. The last feature of each channel is the weighted sum of features filtered by the correlation between the channel and main features. This correlation gets remote semantic dependencies by linking feature maps from different channels, enhances category variability, and improves the semantic information in skip connections. These results are achieved according to changes in the network and improved it. Fig. 5 shows the Dice Coefficient diagram and cost function (loss) over 150 epochs. The close alignment between the training and validation curves, along with their consistent convergence behavior, indicates the absence of significant overfitting and suggests that the model generalizes well to unseen data.

The network prediction consists of two volumes that match the resolution of the original input data. These predictions are processed through a sigmoid layer, which calculates the foreground or background probability for each voxel. In medical images, such as in this study, only a small part of the area of interest is scanned. This can make it difficult for the learning process to understand the local minimum of the loss function. As a result, the network relies on background predictions. Consequently, the foreground area may be overlooked or only partially identified. Previous methods used loss that uses sample weighting. It prioritizes the foreground areas over the background during the learning process. In this article, we use a combination of the Dice coefficient cost function and a binary cost function.

(6) $${Loss=Loss}_{binarycross}+{Loss}_{Dice}$$

The proposed network is fully trained on the BraTS2021 dataset, using images of a resized of 128 × 128 × 128. These images also have ground truth labels annotated manually by trusted experts. To enhance robustness and improve accuracy in the experimental dataset, we augmented the original training data. For data augmentation, we employ random three-dimensional rotations within a range of ±10°, in addition to horizontal and vertical flipping, to increase spatial diversity in the training data. Furthermore, Gaussian noise with a variance of σ = 0.01 is added to enhance model robustness and reduce overfitting. Experimental dataset a total of 1251 patients with MRI images were included, with 876 patients designated for training and the rest allocated for testing and validation. The optimization function used in this study is Adam, which starts with a learning rate of 0.01 and a momentum coefficient of 0.99, which is reduced every 150 epochs by half every 10 epochs.

**Fig. 6:**
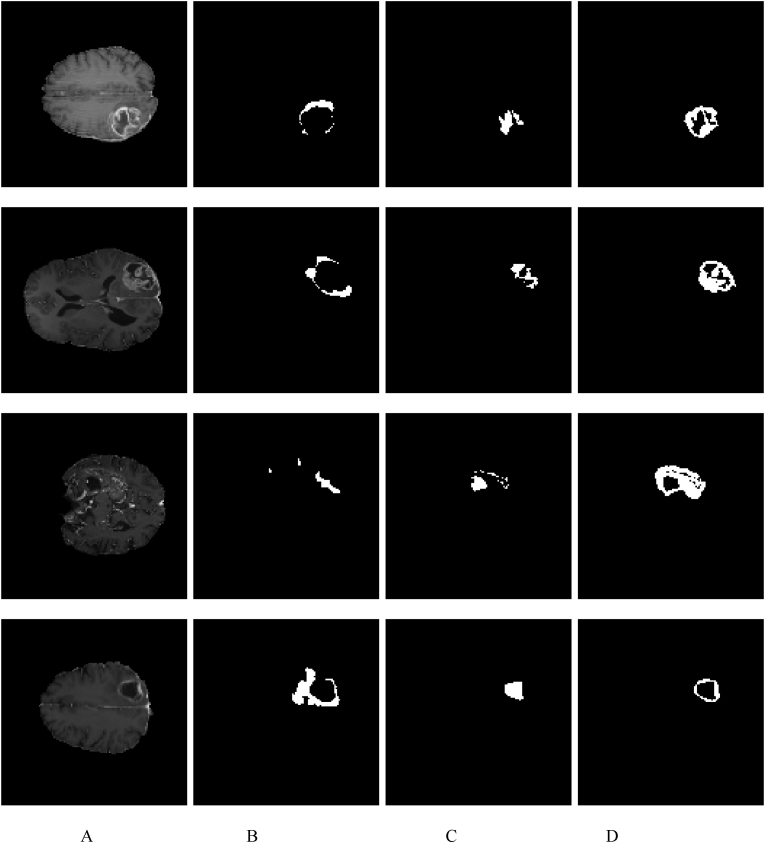
The result of tumor segmentation. A) Original Image B) Enhancing tumor C) Tumor Core D) Whole Tumor.

Fig. 6 illustrates the segmentation outcomes for BraTS2021, classified into three different classes, Enhancing tumor, Whole tumor, and Tumor core. Table 1 presents the results derived from the Dice Coefficient, Sensitivity, and Specificity for train data.

**Table 1:** Result of V-net Model.3.

Train	Whole Tumor	Tumor Core	Enhancing Tumor
**Dice Coefficient**	87	81.2	74.43
**Specifity**	99	98	99
**Sensitivity**	89	82	79

## 4 Discussion

The method in this article effectively addresses the interdependence of channels by separating their features, allowing to suppression of less important channels. After the encoder extracts the features low-resolution and high-resolution feature maps are processed through the Attention Gate module. This method is not affected by tumor size and location. It preserves spatial information and structural composition at different resolutions for MRI images with different intensities. Our model efficiently identifies and segments tumor areas into sub-areas, helping specialists in early diagnosis and treatment. Our results, detailed in Table 2, show strong performance in detecting the Whole tumor area, tumor core, and enhanced areas. Compared to state-of-the-art methods like U-Net and Other V-Net, our improved V-Net with 3D Attention Gate Stands out by achieving a balanced accuracy across different tumor areas, by its novel volumetric attention mechanism. Specifically, the model achieved a Mean Dice Score of 87 for Whole Tumor and 74.43 for Enhancing Tumor, demonstrating its reliability. This is primarily due to the 3D Attention Gate's ability to focus on spatial salience while the dilated convolutions maintain a broad receptive field, crucial for capturing the vast heterogeneity of gliomas without the ‘gridding effect’ often seen in standard CNNs. Unlike many high-parameter models like Swin-UNETR or Mamba-based architectures that demand immense GPU memory, our approach remains computationally feasible, thanks to the integration of Group Normalization which stabilized the training process. While other brain tumor segmentation algorithms such as Mamba-based and dual-branch models demonstrate high segmentation accuracy, depend on extensive preprocessing, our proposed approach emphasizes the extraction of volumetric features through the effective use of dilated convolutions. This design enables a more balanced trade-off between computational efficiency and segmentation performance, particularly for heterogeneous tumor regions, and also reduces such dependencies, suggesting a more adaptable solution for real-time clinical applications. Future work will focus on enhancing segmentation methods and edge detection and extracting more specific tumor information. We found that the dilated convolution (Basic or Large) results can always be improved, and do not overlap with any other post-processing steps. Recent architectures, such as Swin-UNETR (2024) (Sun Yasin et al., 2026) and Mamba-UNet (2025) (Zhu et al., 2025), have reported higher Dice scores of 91.5% and 92.3%, respectively. However, the proposed 3D Attention V-Net emphasizes structural efficiency and computational practicality. By leveraging dilated convolutions, the model attains competitive performance while maintaining a significantly lower parameter count, thereby enhancing its suitability for deployment in resource-constrained clinical environments. While recent architectures such as Mamba-based and Swin-UNETR models demonstrate high segmentation accuracy, the proposed approach emphasizes the extraction of volumetric features through the effective use of dilated convolutions. This design enables a more balanced trade-off between computational efficiency and segmentation performance, particularly for heterogeneous tumor regions.

**Table 2:** The comparison of brain tumor segmentation results for different BraTS data.

Methods	Dataset	Backbone	Whole Tumor	Tumor Core	Enhancing Tumor	Notes
Swin UNetR (Zhang et al., 2025)	BraTS	Transformer	78	19	19	Transformer-based, limited on small datasets
Attention V-Net (Giri et al., 2022)	BraTS	V-Net	80	63.9	53.6	Attention on skip connections
scSE-Nl V-Net (Zhou et al., 2022)	BraTS	V-Net	82	76	65	Channel + spatial squeeze-excitation
Challenges in Brain (Ruffle et al., 2023)	BraTS	Ensemble	**90.7**	70.1	70.1	Handle incomplete imaging data
Cascaded V-Nets (Rui et al., 2020)	BraTS	V-Net	**87.61**	79.53	73.64	Two-stage cascade network
**Proposed Method**	BraTS	V-Net	87	**81.2**	**74.43**	**Residual + Dilated+3D AG**

## 5 Conclusion

Brain tumors are one of the most serious diseases worldwide. It affects more than 20 million people each year. It is responsible for nearly half of all deaths. This study proposed a novel approach to 3D brain tumor segmentation by integrating a V-Net architecture with attention gate, dilated layer, and residual connection. Our proposed model effectively leverages the strengths of these components to enhance feature extraction and improve segmentation accuracy. The proposed 3D improved V-Net with Attention Gate achieved dice score 87%, 81.2%, and 74.43% for whole tumor, tumor core, and enhancing tumor, respectively, outperforming several recent methods while maintaining computational efficiency. This network is made of blocks, and each of these blocks is made of different layers. The dilated convolutions in the network help in learning both local and global information, improving accuracy and adaptability in segmenting tumors. Residual learning methods make it possible to train networks that are much deeper than previously possible and improve efficiency in various performance measurement tasks such as image recognition. The attention gate guides the model to focus on important areas, reducing activation in irrelevant regions. This increases the representative power of the model without significantly increasing the cost computational or parameters. This makes it easy to integrate with various CNN models. Experiment results demonstrate that our approach outperforms existing state-of-the-art methods in terms of segmentation accuracy and robustness across various datasets. The proposed model not only achieves superior performance metrics but also exhibits improved generalization capabilities, making it an important tool for clinical applications in neuroimaging. Future work will focus on further refining the model and exploring its applicability to other types of medical image segmentation tasks. Overall, the core innovation of this study depends on the development of 3D improved V-Net architecture, combined with a 3D Attention Gate that emphasizes volumetric tumor features. This approach not only avoids traditional 2D limitations but also sets a new benchmark for automated segmentation by adapting to the complex 3D structures of brain tumors, preparing for more robust clinical tools.

## Ethical approval

This study was conducted exclusively using publicly available dataset (BraTS2021) and did not involve human participants, animal subjects, clinical procedures, or the collection of identifiable personal data. The research project was registered in the Iranian Research Institute for Information Science and Technology (IranDoc) thesis registration system on November 20, 2023, under registration number **1842150** (available at sabt.irandoc.ac.ir). The study was carried out under the institutional research authorization procedures of the participating universities.

## Funding

No funds, grants, or other support was received.

## CRediT authorship contribution statement

**Sima Esmaeilzadeh Asl:** Conceptualization, Formal analysis, Investigation, Methodology, Writing – original draft, Writing – review & editing. **Mehdi Chehel Amirani:** Formal analysis, Writing – review & editing. **Hadi Seyedarabi:** Conceptualization, Methodology, Supervision, Validation, Writing – review & editing.

## Declaration of competing interest

The authors declare that they have no known competing financial interests or personal relationships that could have appeared to influence the work reported in this paper.

## Data Availability

The data used in this study is publicly available and the information has been provided in the paper.
